# Infectious diseases in the first year of life, perinatal characteristics and childhood acute leukaemia

**DOI:** 10.1038/sj.bjc.6601384

**Published:** 2004-01-06

**Authors:** N Jourdan-Da Silva, Y Perel, F Méchinaud, E Plouvier, V Gandemer, P Lutz, J P Vannier, J L Lamagnére, G Margueritte, P Boutard, A Robert, C Armari, M Munzer, F Millot, L de Lumley, C Berthou, X Rialland, B Pautard, D Hémon, J Clavel

**Affiliations:** 1 Institut National de la Santé et de la Recherche Médicale, INSERM U170-IFR69, 94807 Villejuif, France; 2 Hôpital Pellegrin, Bordeaux, France; 3 Hôtel Dieu. Hôpital mère et enfant, Nantes, France; 4 Hôpital Saint-Jacques, Besançon, France; 5 Hôpital Sud, Rennes, France; 6 Hôpital Civil, Strasbourg, France; 7 Hôpital Charles Nicolle, Rouen, France; 8 CHRU Clocheville, Tours, France; 9 Hôpital Arnaud de Villeneuve, Montpellier, France; 10 Hôpital de la Côte de Nacre, Caen, France; 11 Hôpital d'Enfants, Toulouse, France; 12 Hôpital de la Tronche, Grenoble, France; 13 American Memorial Hospital, Reims, France; 14 Hôpital Jean Bernard, Poitiers, France; 15 Centre Hospitalier Dupuytren, Limoges, France; 16 Centre Hospitalier A Morvan, Brest, France; 17 Centre Robert Debré, Angers, France;; 18 Centre Hospitalier Universitaire, Amiens, France

**Keywords:** childhood, leukaemia, infections, perinatal, epidemiology

## Abstract

The objective of the present study was to investigate the role of early common infections and perinatal characteristics in the aetiology of childhood common leukaemia. A case–control study was conducted from 1995 to 1998 in France, and included 473 incident cases of acute leukaemia (AL) (408 acute lymphoblastic leukaemia (ALL), 65 acute myeloid leukaemia (AML) age-, sex- and region-matched with 567 population-based controls. Data on the medical history of the child and his/her environment were collected using self-administered questionnaires. Analyses were conducted using nonconditional logistic regression. A slight negative association with early infections was observed (OR=0.8; 95% CI (0.6–1.0)). The association was stronger for early gastrointestinal infections. Early day-care was found to be associated with a decreased risk of AL (OR=0.6; 95% CI (0.4–0.8) and OR=0.8; 95% CI (0.5–1.2) for day-care starting before age 3 months and between 3 and 6 months, respectively). No association with breast-feeding was observed, irrespective of its duration. A birth order of 4 or more was associated with a significantly increased risk of AL (OR=2.0; 95% CI (1.1–3.7) with ALL). A history of asthma was associated with a decreased risk of ALL (OR 0.5; 95% CI (0.3–0.90). Although the results regarding birth order and breast-feeding do not fit with Greaves' hypothesis, the study supports the hypothesis that early common infections may play a protective role in the aetiology of childhood leukaemia, although this effect was not more marked for common ALL.

Childhood leukaemia is the most common cancer of childhood and only a few cases can be explained by known risk factors, such as ionising radiation, cancer chemotherapy or Down's syndrome.

Greaves has formulated the hypothesis that delayed exposure to common infections leads to an increased risk of childhood leukaemia, especially common pre-B acute lymphoblastic leukaemia (ALL), which has an incidence peak between ages 2 and 6 years. Childhood ALL is considered to be a rare response to common infections (Greaves, 1988; Greaves and Alexander, 1993; Greaves, 1997). The pathogenesis of leukaemia is believed to occur in two phases. The first genetic event is considered to take place during pregnancy, during the expansion of B-cell precursors. The second genetic event is thought to occur in the same mutant clone, following an immune stress, such as a common infection. The delayed exposure to infection is considered to increase the number of target cells with the ‘first hit’ present at older ages. On the basis of this hypothesis, a child isolated from infectious agents at the beginning of his/her life would be at a higher risk of ALL, while a high birth order value, early common infections and early day-care would be protective factors.

The present study investigated Greaves' hypothesis in a population-based case–control study by analysing the relations between childhood acute leukaemia (AL) and early common infections, day-care attendance and breast-feeding, paying particular attention to ALL. Perinatal characteristics and childhood medical history were also investigated.

## SUBJECTS AND METHODS

### Subjects

A population-based case–control study was conducted from 1995 to 1998. Cases were derived from the National Registry of Childhood Leukaemia and Lymphoma (NRCL), which registers all the cases of leukaemia among children less than 15 in mainland France since 1990. Thus, to be eligible in the study, cases were required to be under 15 years old and be a resident in mainland France at the time of diagnosis. In addition, the mother had to be able to fill out a questionnaire and the doctor had to authorise contact with the mother. Cases in four regions that were already involved in a hospital-based case–control study (Perrillat *et al*, 2002a, 2002b), and the cases in four other regions in which the oncology department could not contribute to the study for practical reasons were excluded.

During the period 1995–1998, the NRCL registered 786 cases of AL in the 14 regions. Of those cases, 646 were eligible and 140 were not eligible: 25 were not known at the time of the study, two were known not to have parents, three had parents who were unable to fill out the questionnaire for linguistic (1) or social (2) reasons, 110 were too sick for their parents to be interviewed (28 of them died before the physician could pass on the questionnaire). The serious condition of the cases was a particular reason for noneligibility before the age of 1 year, when 17 out of the 33 registered cases were eligible. The overall participation rate, relative to all registered cases, was thus 60.2% (473 out of 786), and the response rate was 73.2% (473 out of 646).

The controls were randomly selected from the general population with stratification respecting the age, gender and regional distribution of the cases. Both the case and control mothers completed a self-administered questionnaire, distributed by the child's physician for cases, and by mail for controls. Controls were randomly selected using age, sex and region quotas from a sample of 30 000 phone numbers representative of the French population with respect to area of residence and municipality size categories. The control distribution was determined *a priori*, on the basis of the expected age, sex and region distribution of the cases derived from the previous years of registration. The study was designed with the same number of cases as controls with a frequency matching on age, sex and region.

A total of 805 controls were eligible. The mothers of 574 controls completed the self-administered questionnaire. Five controls were excluded because they were adopted and two because the questionnaires had too many missing values (only the first page, i.e. circumstances of birth, was completed). Thus, a total of 567 controls were included in the study. The response rate was 71% for the controls.

### Data collection

On average, the questionnaire was completed 10 months after the diagnosis (before 6 months for 212 cases, between 6 and 12 months for 113 cases, between 12 and 18 months for 76 cases, after 18 months for 72 cases). It was completed within 6 months for the controls.

Details on the diagnosis of leukaemia were collected from the medical records by the Registry investigators. Data on the perinatal period, child's medical history and environment were collected using a standardised self-administered questionnaire. The questions on medical history were closed questions. The data on early infections included the history of ear, nose or throat (ENT), gastrointestinal (GI) and other infections, and the frequency of each type of infection (⩾1 per month; <1 per month and ⩾1 per quarter; <1 per quarter and ⩾1 per year, less often) for the age groups: <1; 1–2; 3–4; 5 years and more.

The data on factors promoting infections included birth order of the index child, duration of breast-feeding, and history of day-care attendance.

### Statistical analysis

Odds ratios (OR) were estimated using an unconditional logistic regression model including stratification variables, that is, gender, age and region, using the SAS® software package.

The analyses of day-care attendance, early infections and breast-feeding were restricted to children aged over 1 year in order to be certain that infections before age 1 had already taken place in both the cases and the controls.

The children with Down's syndrome (10 cases and two controls) were excluded from most of the analyses.

## RESULTS

The cases and controls were very similar with respect to age, gender and region of residence at the time of diagnosis (Table 1

**Table 1:** Sample description for the cases and controls

	**Cases (%) (*N*=473)**	**Controls (%) (*N*=567)**	***P***
*Gender*			NS
Male	260 (55)	326 (57)	

*Age at diagnosis (years)*			NS
0–1	45 (10)		
2–3	115 (24)	78 (14)	
4–5	110 (23)	148 (26)	
6–9	113 (24)	109 (19)	
10–15	90 (19)	130 (23)	
		102 (18)	
*Region of residence at diagnosis*			NS
Alsace	28 (6)	29 (5)	
Aquitaine	59 (12)	42 (7)	
Bretagne	45 (10)	70 (12)	
Centre	51 (11)	62 (11)	
Champagne Ardennes	22 (5)	33 (6)	
Franche-Comté	28 (6)	31 (5)	
Languedoc-Roussillon	31 (7)	48 (8)	
Limousin	8 (2)	14 (2)	
Midi-Pyrénées	33 (7)	42 (7)	
Basse-Normandie	29 (6)	28 (5)	
Haute-Normandie	29 (6)	27 (5)	
Pays de Loire	48 (10)	75 (13)	
Picardie	31 (7)	36 (6)	
Poitou-Charentes	31 (7)	30 (5)	

*Socioprofessional categories*	NS		
Without employment	15 (3)	8 (1)	
Craftsmen and factory workers	132 (28)	118 (21)	
Farmers and agricultural workers	14 (3)	29 (5)	
Sales and service workers	49 (10)	80 (14)	
Administrative employees	66 (14)	105 (19)	
Intermediate profession	95 (20)	104 (19)	
Intellectual and scientific jobs	55 (12)	64 (11)	
Managers	43 (9)	54 (10)	
X^a^	4	5	

*Maternal education*			NS
<high school	337 (74)	415 (74)	
>high school	120 (26)	144 (26)	
X^a^	16	8	

*Paternal education*			NS
<high school	337 (77)	424 (79)	
>high school	102 (23)	113 (21)	
X^a^	34	30	

a Missing values.

). In total, 48% of the cases and 45% of the controls contributed to the age groups 2–3 and 4–5 years, corresponding to the peak of incidence of leukaemia, and 12 cases (3%) and 35 controls (6%) were younger than 1 year.

There was no difference between the cases and controls with respect to the distribution of parental socioprofessional category, or maternal or paternal educational level.

No association between childhood leukaemia and birth weight, term of pregnancy, maternal age at birth and history of previous foetal losses was observed (Table 2

**Table 2:** Perinatal characteristics and childhood leukaemia

	**ALL**				**AML**			
	**Cases (*N*=408)**	**Controls (*N*=567)**	**OR^a^**	**95% CI**	**Cases (*N*=65)**	**Controls (*N*=567)**	**OR^a^**	**95% CI**
*Mean birth weight (g)*								
Mean	3322	3314			3294	3314		
s.d.^b^	(514)	(522)			(396)	(522)		

*Birth weight (g)*								
<2500	22	31	1.0	(0.6–1.9)	1	31	0.3	(0.03–2.1)
2500–2999	73	96	1.1	(0.7–1.6)	12	96	0.9	(0.4–1.9)
3000–3499	160	217	1.0	Ref	32	217	1.0	Ref
3500–3999	97	172	0.8	(0.6–1.1)	14	172	0.6	(0.3–1.2)
⩾4000	40	37	1.4	(0.8–2.3)	3	37	0.8	(0.2–2.8)
X^c^	16	14			3	14		

*Term of pregnancy*								
<37	41	45	1.4	(0.9–2.2)	5	45	1.4	(0.5–4.1)
37–38	80	144	0.7	(0.5–1.0)	13	144	1.0	(0.5–2.1)
39–40	220	304	1.0	Ref	32	304	1.0	Ref
⩾41	32	52	0.9	(0.5–1.5)	7	52	1.3	(0.5–3.3)
X^c^	35	22			8	22		

*Birth order*								
1	180	267	1.0	Ref	35	267	1.0	Ref
2	131	199	0.9	(0.7–1.2)	12	199	0.5	(0.2–1.0)
3	64	73	1.1	(0.8–1.7)	12	73	1.3	(0.6–2.8)
4+	32	22	2.0	(1.1–3.7)	5	22	2.5	(0.8–7.5)
X^c^	1	6			1	6		
	*P-trend* =*0.07*				*P-trend*>*0.10*			

*Maternal age at birth*								
<25	84	93	1.4	(1.0–2.1)	11	93	0.9	(0.4–2.0)
25–29	154	241	1.0	Ref	30	241	1.0	Ref
30–34	107	169	0.9	(0.7–1.3)	14	169	0.7	(0.3–1.3)
⩾35	57	56	1.6	(1.0–2.5)	8	56	1.3	(0.5–3.1)
X^c^	6	8			2	8		

*Previous foetal losses*								
0	313	436	1.0	Ref	49	436	1.0	Ref
1	74	88	1.2	(0.8–1.7)	11	88	1.1	(0.5–2.3)
⩾2	16	28	0.8	(0.4–1.6)	2	28	0.6	(0.1–2.8)
X^c^	5	15			3	15		

*Down's syndrome*								
No	400	565	1.0	Ref	63	565	1.0	Ref
Yes	8	2	4.4	(0.9–2.2)	2	2	11.7	(1.3–108.5)

a Adjusted for stratification variables (gender, age at diagnosis, region of residence at diagnosis).

b s.d.=standard deviation.

c X=missing values.

).

A statistically significant association between birth order and childhood ALL was observed (*P*-trend=0.07–OR=2.0; CI (3.1–3.7) for children born fourth). A similar association was observed with AML.

No association between breast-feeding, irrespective of its duration, and childhood AL was observed (Table 3

**Table 3:** Association between childhood acute leukaemia and breast-feeding (analysis restricted to children older than 1 year)

	**ALL**				**AML**			
	**Cases (*N*=393)**	**Controls (*N*=530)**	**OR^a^**	**95% CI**	**Cases (*N*=59)**	**Controls (*N*=530)**	**OR^a^**	**95% CI**
*Breast-feeding*								
No	216	307	1.0	Ref	29	307	1.0	Ref
Yes	176	222	1.1	(0.9–1.5)	30	222	1.4	(0.8–2.5)
X^b^	1	1			0	1		

*Breast-feeding duration*								
0	216	306	1.0	Ref	29	306	1.0	Ref
<3 months	86	105	1.2	(0.8–1.7)	14	105	1.4	(0.7–2.9)
3–6 months	57	75	1.1	(0.7–1.6)	12	75	1.7	(0.8–3.7)
>6 months	29	29	1.4	(0.8–2.5)	2	29	0.5	(0.1–2.1)
X^b^	5	15			2	15		

a ORs adjusted for stratification variables: gender, age at diagnosis, region of residence at diagnosis.

b X=missing values.

).

The results for early infections are shown in Table 4

**Table 4:** Association between childhood acute leukaemia and common early infections (analysis restricted to children older than 1 year)

	**ALL**				**AML**			
	**Cases (*N*=393)**	**Controls (*N*=530)**	**OR^a^**	**95% CI**	**Cases (*N*=59)**	**Controls (*N*=530)**	**OR^a^**	**95% CI**
⩾*four infections in the 1st year of life*								
No	230	309	1.0	Ref	31	309	1.0	Ref
Yes	104	172	0.8	(0.6–1.0)	18	172	1.4	(0.7–2.6)
X^b^	59	49			10	49		

⩾*4 ENT*^c^ *infections in the 1st year of life*								
No	257	340	1.0	Ref	35	340	1.0	Ref
Yes	101	166	0.8	(0.6–1.1)	17	166	1.3	(0.7–2.4)
X^b^	35	24			7	24		

⩾*4 GI*^d^ *infections in the 1st year of life*								
No	342	456	1.0	Ref	47	456	1.0	Ref
Yes	2	18	0.1	(0.03–0.6)	0	18	—	
X^b^	49	56			12	56		

a ORs adjusted for stratification variables: gender, age at diagnosis, region of residence at diagnosis.

b X=missing values.

c ENT=ear, nose throat.

d GI=gastrointestinal.

. The mothers of 122 cases (104 ALL and 18 AML) and of 172 controls declared at least four common infections in the first year of their child's life. ENT infections were highly predominant, while infections other than ENT were reported at lower frequency: 44 cases and 52 controls reported only one GI infection during the first year and 15 cases and 26 controls reported only one infection other than ENT or GI during the first year. A statistically significant negative association between common infections before age 1 year and childhood ALL (OR=0.8; CI (0.6–1.0)) was observed. This association was not observed with AML. The association was stronger for early GI infections (OR=0.1; CI (0.03–0.6)), but this finding was based on only two cases and 18 controls.

In order to evaluate the potential influence of the missing values on the results presented in Table 4, we also estimated the OR associated with total ENT or GI infections either by including the missing values for cases and controls in the category of the least infected children or by including the missing values of cases and controls in the category of the most frequently infected children. The OR remained significantly less than 1 if missing values were assigned to the unexposed group, and increased at most to 1.0 if, conversely, the missing values were assigned to the group of children who had more than four infections in their first year of life.

Taken as a whole, day-care attendance was associated with childhood AL (OR=0.7; CI (0.6–1.0) for ALL) as shown in Table 5

**Table 5:** Association between childhood acute leukaemia and day-care attendance (analysis restricted to children older than 1 year)

	**ALL**				**AML**			
	**Cases (*N*=393)**	**Controls (*N*=530)**	**OR^a^**	**95% CI**	**Cases (*N*=59)**	**Controls (*N*=530)**	**OR^a^**	**95% CI**
*Day-care*								
No	220	259	1.0	Ref	34	259	1.0	Ref
Yes	167	266	0.7	(0.6–1.0)	24	266	0.8	(0.5–1.5)
X^b^	6	5			1	5		

*Type of day-care*								
None	220	259	1.0	Ref	34	259	1.0	Ref
Nurse only	120	198	0.7	(0.5–1.0)	17	198	0.8	(0.4–1.6)
Full time day-care centre only	29	34	1.1	(0.6–1.8)	5	34	1.3	(0.4–3.7)
Occasional day-care centre only	6	15	0.4	(0.2–1.2)	1	15	0.7	(0.1–6.4)
More than one type of day-care	12	19	0.7	(0.3–1.5)	1	19	0.6	(0.1–5.1)
X^b^	6	5			1	5		

*Age at start of day-care (nurse or day-care centre)*								
<3 months	61	120	0.6	(0.4–0.8)	12	120	1.0	(0.5–2.2)
3–6 months	53	82	0.8	(0.5–1.2)	6	82	0.6	(0.2–1.5)
6–12 months	25	25	1.2	(0.6–2.1)	4	25	1.3	(0.4–4.1)
⩾12 months	21	25	1.0	(0.5–1.8)	1	25	0.3	(0.04–2.8)
Never	220	259	1.0	Ref	34	259	1.0	Ref
X^b^	13	19			2	19		
			*P-trend*<*0.05*					

*Age at start of full-time day-care centre*								
Never	348	475	1.0	Ref	52	475	1.0	Ref
1–3 months	10	23	0.6	(0.3–1.3)	3	23	1.5	(0.4–5.6)
4–6 months	9	10	1.4	(0.5–3.5)	3	10	2.5	(0.6–10.2)
7–12 months	8	6	2.1	(0.7–6.2)	0	6		
⩾12 months	11	9	1.6	(0.6–4.0)	0	9		
X^b^	7	7			1	7		

a ORs adjusted for stratification variables: gender, age at diagnosis, region of residence at diagnosis.

b X=missing values.

. The association was only observed when day-care started before age 6 months (OR=0.6; CI (0.4–0.8) for age less than 3 months; OR=0.8; CI (0.5–1.2) for age 3–6 months). The trend with respect to the age of starting day-care was statistically significant.

After exclusion of children with Down's syndrome, 221 cases (210 ALL and 11 AML) and 255 controls belonged to the 2–6 years age bracket corresponding to the incidence peak, while 199 cases (161 ALL and 38 AML) and 232 controls were 6 years old or more. Of the 393 ALL after age one, 304 were of the common B-cell type, 54 were of the T-cell type, and four were B mature.

Data were also analysed separately depending on the age at diagnosis (2–6 years *vs* older) and on the subtype of ALL (common B-cell *vs* other ALL). The association between early frequent common infections and AL was restricted to the 2–6 years age group, but not to the common B-cell ALL subtype (Table 6

**Table 6:** Association between childhood acute leukaemia and factors implicated in Greaves' hypothesis, according to age at diagnosis and ALL subtype

	**Age**				**ALL subtype^a^**			
	**2–6 years**		**6–15 years**		**Common B-cell ALL**		**Other ALL**	
	**OR^b^**	**95% CI**	**OR^b^**	**95% CI**	**OR^b^**	**95% CI**	**OR^b^**	**95% CI**
⩾*4 infections in the 1st year of life*								
Any infection	0.7	(0.5–1.0)	1.0	(0.6–1.6)	0.8	(0.6–1.1)	0.7	(0.4–1.1)
ENT	0.7	(0.5–1.0)	1.0	(0.6–1.6)	0.8	(0.6–1.1)	0.7	(0.4–1.2)
Digestive	0		0.5	(0.1–2.8)	0.1	(0.01–0.7)	0.3	(0.04–2.5)
*Day-care*								
Yes *vs* no	0.8	(0.5–1.2)	0.7	(0.5–1.1)	0.8	(0.6–1.0)	0.5	(0.3–0.9)

*Age at start of day-care (any type)*								
<3 months	0.6	(0.4–1.0)	0.7	(0.4–1.2)	0.6	(0.4–0.9)	0.4	(0.2–0.8)
3–6 months	0.9	(0.5–1.6)	0.8	(0.4–1.3)	0.8	(0.6–1.3)	0.5	(0.2–1.1)
>6 months	1.3	(0.7–2.4)	0.7	(0.4–1.6)	1.1	(0.7–1.8)	1.0	(0.5–2.1)
Never	1.0	Ref	1.0	Ref	1.0	Ref	1.0	Ref

*Birth order*								
1	1.0	Ref	1.0	Ref	1.0	Ref	1.0	Ref
2	0.8	(0.6–1.3)	0.7	(0.4–1.1)	0.9	(0.6–1.2)	1.1	(0.6–1.8)
3	1.4	(0.8–2.4)	0.7	(0.4–1.3)	1.2	(0.8–1.9)	0.8	(0.4–1.7)
⩾4	1.5	(0.7–3.3)	2.4	(0.9–6.3)	2.0	(1.1–3.8)	2.3	(0.9–6.1)

*Breast-feeding*								
Yes *vs* no	1.0	(0.7–1.5)	1.4	(0.9–2.0)	1.2	(0.9–1.5)	1.1	(0.7–1.8)
*Breast-feeding duration*								
Never	1.0	Ref	1.0	Ref	1.0	Ref	1.0	Ref
<3 months	0.8	(0.5–1.3)	1.7	(1.0–2.9)	1.2	(0.8–1.7)	1.1	(0.6–1.9)
⩾3 months	1.4	(0.8–2.2)	1.0	(0.6–1.6)	1.2	(0.8–1.7)	1.3	(0.7–2.2)

a Analysis restricted to children older than 1 year.

b ORs adjusted for stratification variables: gender, age at diagnosis, region of residence at diagnosis.

). The associations with day-care were specific neither to the common B cell ALL subtype nor to the age bracket 2–6 years.

As shown in Table 7

**Table 7:** Medical history of asthma or infantile diseases and risk of childhood acute leukaemia

	**ALL**				**AML**			
	**Cases (*N*=400)**	**Controls (*N*=565)**	**OR^a^**	**95% CI**	**Cases (*N*=63)**	**Controls (*N*=565)**	**OR^a^**	**95% CI**
*Asthma*								
Asthma	17	44	0.5	(0.3–0.9)	2	44	0.4	(0.1–1.7)
Asthma and/or bronchodilators (BD)								
No asthma and no BD	358	501	1.0	Ref	59	501	1.0	Ref
Asthma or BD	25	31	1.0	(0.6–1.8)	2	31	0.7	(0.1–3.0)
Asthma and BD	6	29	0.3	(0.1–0.7)	1	29	0.3	(0.03–2.3)
X^b^	11	4			1	4		

*Infantile diseases before the diagnosis*								
Measles	41	38	1.7	(1.0–2.9)	8	38	1.3	(0.5–3.1)
Rubella	37	24	2.4	(1.4–4.1)	3	24	1.1	(0.3–3.9)
Mumps	24	20	1.9	(1.0–3.6)	6	20	1.6	(0.6–4.5
Chickenpox	220	323	0.9	(0.6–1.2)	33	323	0.7	(0.3–1.4)
Any of the above infantile diseases								
None	147	222	1.0	Ref	26	222	1.0	Ref
1	178	272	0.9	(0.7–1.3)	24	272	0.7	(0.3–1.6)
2 or more	53	50	1.6	(1.0–2.8)	7	50	0.7	(0.2–2.1)

a ORs adjusted for stratification variables: gender, age at diagnosis, region of residence at diagnosis.

b X=missing values.

, a statistically significant negative association between asthma and childhood ALL was observed (OR=0.5; CI (0.3–0.9)). The association was even stronger for asthmatic children regularly treated with bronchodilators (OR=0.3; CI (0.1–0.7)).

No significant association with a history of chickenpox, mumps or glandular fever was observed (Table 7). In contrast, ALL was associated with a history of measles (OR=1.7; CI (1.0–2.9)) and rubella (OR=2.4; CI (1.4–4.1)). A history of viral hepatitis was only reported for two cases and one control (OR=3.3; CI (0.3–37.0)).

## DISCUSSION

One of the main objectives of the present population-based study was to test whether early common infections were associated with a reduced risk of AL. Slight negative associations between ALL and common early infections and day-care were observed. No association with breast-feeding, irrespective of duration, was observed. A birth order of four or more was associated with an increased risk of AL. A history of two or more infantile viral diseases was positively associated with ALL while a history of asthma was negatively associated with ALL.

The data were collected from a standardised self-administered questionnaire. The response rates for the cases and controls were very similar and the nonrespondent controls did not differ from the respondent controls in terms of age, gender or region of residence. The percentage mortality rates, obtained from the NRCL, for the respondent and nonrespondent cases were very similar (12 and 8%, respectively) making a strong survival bias unlikely. The exhaustiveness of the NRCL is close to 99%, making unlikely a selection through the process of cases' identification.

Recalling common infections may be difficult, and this may explain the rather high number of missing values in the questionnaires. However, when the missing values were assigned either exposed or unexposed status (with the hypothesis of a nondifferential bias), the OR remained less than unity. An OR of 1.0 was only obtained when all the missing values were classed as ‘most often infected’, which is very unlikely. Case mothers may have declared minor health problems less often, and thus introduced a differential recall bias. However, the self-administered questionnaire contained closed and very precise questions. Over-reporting by control mothers was possible, but unlikely. Conversely, a nondifferential recall bias was more likely, given that the questionnaire was self-administered, and may have reduced the association with common early infections.

Few papers have addressed the role of early common infections yet. All but one (Dockerty *et al*, 1999) found a negative association with early common infections. Van Steensel-Moll *et al* (1986) observed a significant negative association between common colds before age 1 year and the risk of ALL. Neglia *et al* (2000) observed a significant trend towards a reduction in the risk of AL with an increase in the number of episodes of otitis before age 1 year. The trend was stronger for ALL. Perrillat *et al* (2002b) found a negative association between the risk of ALL and a history of four or more episodes of otitis before age 2 years, on the one hand, and a history of ENT surgical procedures before age 2 years, on the other hand.

Day-care attendance can be considered a surrogate of early contact with infections. In our study, day-care was slightly negatively associated with ALL when initiated early. However, curiously, the association did not concern full-time day-care centres. All the authors who have studied the association between day-care and leukaemia have reported OR of less than one (Petridou *et al*, 1993, 1997; Neglia *et al*, 2000). However, only four studies found significant negative associations (Infante-Rivard *et al*, 2000; Rosenbaum *et al*, 2000; Ma *et al*, 2002; Perrillat *et al*, 2002b). When age at the start of day-care was studied, the negative association was stronger for the youngest.

With regard to breast-feeding, recall bias is difficult to imagine since the mother was asked to indicate the duration of breast-feeding. The questionnaire did not distinguish between mixed feeding and complete breast-feeding. Over-reporting of long-duration breast-feeding by case mothers cannot be ruled out since some mothers may experience feelings of guilt with regard to breast-feeding. However, such a recall bias is likely to concern mothers who only breastfed for a short duration rather than those who did not breast-feed at all, and is probably insufficient to explain the absence of a negative association. Confounding by birth order is possible, since the older children were less often breastfed and received breast-feeding for shorter durations than the controls. However, adjustments for birth order did not modify the association and there was no interaction between the two variables. The majority of the studies investigating breast-feeding have found a negative association with childhood AL (Davis *et al*, 1988; Petridou *et al*, 1997; Infante-Rivard *et al*, 2000; Rosenbaum *et al*, 2000), which was more marked for prolonged breast-feeding (Magnani *et al*, 1988; Dockerty *et al*, 1999; Shu *et al*, 1999; Smulevich *et al*, 1999; Bener *et al*, 2001; Hardell and Dreifaldt, 2001; Perrillat *et al*, 2002a).

The positive association with birth order observed in the present study was unexpected and did not seem to be explained by sociodemographic characteristics or by the other variables under study. Control mothers with the largest families may have been counter-selected, for instance, because they would have been less available to answer the questionnaire. Although first-born status was included in Greaves' hypothesis as a risk factor, many studies have not observed any association between birth order and ALL or AL (Kaye *et al*, 1991; Petridou *et al*, 1993, 1997; Roman *et al*, 1997; Westergaard *et al*, 1997; McKinney *et al*, 1999; Shu *et al*, 1999; Neglia *et al*, 2000). Only three authors have observed a significant negative association between the risk of leukaemia (or ALL) and birth order (Van Steensel-Moll *et al*, 1986; Schuz *et al*, 1999a; Dockerty *et al*, 2001), in line with Greaves' hypothesis. In contrast, three studies have found a significant positive association between the risk of AL and birth order (Savitz and Ananth, 1994; Infante-Rivard *et al*, 2000; Shu *et al*, 2002).

The negative relation with asthma and bronchodilators may be fortuitous. However, several authors have already pointed out the possibility of a negative association with asthma and other allergic diseases (Magnani *et al*, 1990; Petridou *et al*, 1997; Schuz *et al*, 1999b; Wen *et al*, 2000), and the association deserves further investigation.

In conclusion, although the results regarding birth order and breast-feeding do not fit with Greaves' hypothesis, the study supports the hypothesis that early common infections may play a protective role in the aetiology of childhood leukaemia, although this effect was not more marked for common ALL.
